# HBx Drives Liver Cancer Stem Cell Generation Through Stimulating Glucose Metabolic Reprogramming

**DOI:** 10.1111/jcmm.70722

**Published:** 2025-07-27

**Authors:** Jinchen Liu, Xueqin Wu, Qiushi Yin, Luying Zhang, Kun Liu, Kailin Huang, Junnv Xu, Xiaowei Li, Bo Lin, Mingyue Zhu, Mengsen Li

**Affiliations:** ^1^ Key Laboratory of Tropical Translational Medicine, Ministry of Education, and Hainan Provincial Key Laboratory of Carcinogenesis and Intervention Hainan Medical University Haikou Hainan Province PR China; ^2^ Department of Medical Oncology The Second Affiliated Hospital of Hainan Medical University Haikou Hainan Province PR China

**Keywords:** cancer stem cells, glucose metabolism reprogramming, HBx, hepatocellular carcinoma, Warburg effect

## Abstract

Recurrence of hepatocellular carcinoma (HCC) is closely related to the infection of hepatitis B virus (HBV). The HBV x protein (HBx) plays a key role in promoting the malignant transformation of hepatocytes and cancer heterogeneity, but the role of HBx in metabolism influencing the generation of cancer stem cells (CSCs) is still unclear. This study explores HBx‐induced glucose metabolic reprogramming of HCC cells to promote the generation of CSCs. Immunohistochemical analysis of the expression of glucose metabolic reprogramming‐related enzymes and stemness markers in HCC tissues and corresponding paracancer tissues of 30 patients; Western blotting, laser confocal microscopy, and metabolism‐detection kits were applied to analyse the expression of glucose metabolism‐related enzymes and cancer stemness markers and glucose metabolic products; the generation of CSCs was observed by stem cell pellet and soft agar colony formation experiments. Results indicated that the expression of PKM2, HK2, LDHA, CSC‐related proteins, and CD133 and CD44 in HCC tissues was significantly higher than that in the corresponding paracancerous tissues. HBx stimulated the expression of the key enzyme of the Warburg effect and CSC‐related proteins, and these proteins were significantly reduced after interference with the expression of the PKM2 protein. PKM2 and OCT4 interact in HCC cells, and PKM2 has a regulatory effect on OCT4 function. This study found that HBx stimulated the Warburg effect and induced HCC stemness reprogramming by activating the PI3K/AKT signalling pathway; PKM2 played a key role in promoting the initiation of HCC stem cells. Targeting HBx and PKM2 is a new strategy for the treatment of HCC.

**Abbreviations**
AFP: alpha‐fetoprotein
ATP: adenosine triphosphate
CSCs: cancer stem cells
EpCAM: epithelial cellular adhesion molecule
GAPDH: glyceraldehyde 3‐phosphate dehydrogenase
GLUTs: glucose transporters
HBx: hepatitis B virus x protein
HCC: hepatocellular carcinoma
hESCs: human embryonic stem cells
HK2: Hexokinase2
LCSCs: liver cancer stem cells
LDH: lactate dehydrogenase
LDHA: lactate dehydrogenase A
mTOR: mammalian target of rapamycin
PI3K/Akt: phosphatidylinositol 3‐kinase/protein kinase B
PKM2: pyruvate kinase M2

## 1 Introduction

Hepatocellular carcinoma (HCC) is the sixth most common cancer and is one of the deadliest cancers. In 2018, the GLOBOCAN database predicted a significant increase (> 5%) in HCC incidence and mortality by 2020 in most countries, accounting for 46.8% and 40.4%, respectively [1]. HCC is one of the top three cancer causes of death in 46 countries worldwide. Another 90 countries had one of the top five causes of death. The GLOBOCAN 2020 database also predicts and reports that 1.4 million people may be diagnosed with HCC in 2040, and 1.3 million people will die from HCC (an increase of 56.4% over 2020) [2, 3]. Hepatitis B virus (HBV) infection causes more than half of liver cancer cases worldwide, and Asia has the highest incidence of HBV aetiology [4, 5]. When the liver is continuously infected with HBV, many different genetic and molecular pathways are altered. Therefore, the liver tissue microenvironment undergoes complex changes, which are also a risk factor for the development of HCC. Although HCC patients infected with HBV are treated with intraductal radiofrequency ablation (RFA), transarterial chemoembolization (TACE), tyrosine kinase inhibitors (TKIs) and immunotherapy are also accompanied by the use of anti‐viral drugs, HCC recurrence, cancer dormancy, and treatment resistance still occur after successful chemotherapy and/or radiotherapy [6, 7]. Due to the abundant and complex cell composition in cancer tissues and the different functions and characteristics of these cells, the sensitivity to clinical treatment is also very different, and this has become a new research direction of drug resistance. Many researchers have reported that tumour heterogeneity is related to the drive of cell subpopulations with stem cell or progenitor cell characteristics, which is the source of cancer recurrence and treatment resistance. Because these cell subpopulations contain characteristics of stem cells with the ability to self‐renew and differentiate, as well as the properties of regenerating malignant behaviours of cancers, these cells are called cancer stem cells (CSCs) [8, 9].

The HBV genome is a partially double‐chained molecule with a relaxed shape in the virus particle, with four open reading frames (ORF), in which the HBV x protein encoded by the x gene (code HBV x protein, HBx) plays a crucial role in the pathogenesis of HBV infection and the replication and transcription of the virus in hepatocytes [10]. HBx can support the virus in terms of gene expression and replication and reduces the ability of immune cells to recognise and clear infected liver cells [11]. HBx causes chronic liver disease (CLD) by infecting the cytoplasm of hepatocytes and stimulating the expression of proinflammatory and cancer‐promoting signal transduction pathways, thus leading to CLD. Therefore, hepatocytes gradually progress into liver cancer with the development of viral infections and the disease course [12]. In the cell nucleus, HBx affects viral and host gene expression by binding to other proteins, altering the activity of transcription complexes, and affecting the expression of epigenetic regulators, such as histone deacetylase and DNA methyltransferase [13]. Drug resistance caused by tumour heterogeneity has always been a problem in clinical treatment. The emergence of CSCs has brought new clinical research directions, and HCC is a malignant tumour with highly heterogeneous characteristics. Liver cancer stem cells (LCSCs) provide a new perspective for research on the treatment and pathogenesis of liver cancer. LCSCs also have characteristics similar to those of normal stem cells because they are poorly differentiated cancer cell subsets and can grow, regenerate and invade tissues [14, 15]. Although there are no highly specific markers that can be used to distinguish HCC cells from LCSCs at this stage, many studies have reported that the differential expression of EpCAM (CD326), CD90, CD44 and CD133 could reflect the difference between LCSCs and HCC cells [16, 17, 18, 19, 20]. LCSCs can also regulate stemness characteristics through multiple stemness reprogramming factors, such as OCT4, SOX2, NANOG, KLF4 and c‐MYC, to promote the generation of LCSCs.

During the development of HCC, owing to the state of hypoxia occurring within the tumour, HCC undergoes metabolic reprogramming to adapt to changes in the tumour microenvironment (TME). The Warburg effect is a new understanding in which cancer cells transform into the glycolytic mode to adapt to survival when the tumour is in a hypoxic environment. However, even under conditions where the supply of oxygen is abundant, cancer cells continue this process and change their energy metabolism from oxidative phosphorylation to aerobic glycolysis. Many studies have found that the key rate‐limiting enzymes of glycolysis, pyruvate kinase M2 (PKM2) and lactate dehydrogenase (LDH), during the course of HCC development, the expression of LDH A (LDHA), glucose transporters (GLUTs), hexokinase 2 (HK2), and pyruvate dehydrogenase kinase (PDK) were up‐regulated. These changes are able to promote the adaptation of cancer cells to a hypoxic environment to maintain the growth and invasion of HCC cells [21, 22].

Previous studies have found a close relationship between HBx, PI3K/Akt/mTOR, the Warburg effect, and stemness reprogramming of LCSCs [23, 24], which stimulates the expression of alpha‐fetoprotein (AFP) to activate PI3K/Akt/mTOR, leading to the initiation of LCSCs [25, 26]. Therefore, we speculated that HBx might regulate the Warburg effect by activating the PI3K/Akt/mTOR signalling pathway and affect the occurrence and development of LCSCs. In this study, we explored the influence of HBx on the Warburg effect and its regulatory effect on the generation of LCSCs. This provides a new strategy to inhibit HBV infection in favour of targeted therapy for liver cancer.

## 2 Materials and Methods

### 2.1 Patients and Specimens

In this study, liver cancer tissues and corresponding paracancerous tissues of 30 patients with HCC were collected from January 2021 to December 2022 at the Second Affiliated Hospital of Hainan Medical University. Paracancerous tissues were defined as those ≥ 2 cm from the edge of the cancerous lesions. All tissue samples were packaged and soaked in 4% paraformaldehyde, made into tissue chip wax blocks using special instruments, and analysed by immunohistochemistry. Liver cancer tissues and corresponding paracancerous tissues of eight patients with liver cancer were collected from May 2023 to December 2023 at the First Affiliated Hospital of Hainan Medical University. Paracancerous tissues were defined as tissues ≥ 2 cm from the edge of the cancerous lesions. After all tissue samples were removed, one group was divided into an internal rotating cryostorage tube, marked with patient information, and frozen in liquid nitrogen, which could be used to extract histamine or RNA for analysis. The other group was removed and divided into centrifuge tubes containing 4% paraformaldehyde, stored at room temperature, and made into tissue wax blocks for immunohistochemical analysis. All study subjects provided informed consent, and the collection and use of tissue specimens was approved by the Medical Ethics Committee of the First Affiliated Hospital and the Second Affiliated Hospital of Hainan Medical University (Ethical Licence number: LW2019312). This study involved the detection of protein expression in human liver cancer samples, strictly in accordance with the Declaration of Helsinki. The research content and process of the project followed international and national ethical requirements for biomedical research.

### 2.2 Immunohistochemical Analysis

The expression and distribution of PKM2, LDHA, SOX2, c‐MYC, CD44 and CD133 in liver cancer tissues were detected by immunohistochemistry. The 5 μm thick paraffin sections were dewaxed and rehydrated according to standard procedures, and the locations of the tissue chip extraction points were determined by staining the nuclei and cytoplasm with haematoxylin and eosin, respectively. The tissue chip fusion device was then used to fuse the tissue points and wax blocks. The two must be completely fused to successfully create tissue‐chip wax blocks. Heat induction in sodium citrate buffer causes re‐exposure of the antigenic determinant. 3% H_2_O_2_ inhibited endogenous peroxidase, and 3% bovine serum albumin (BSA) blocking solution blocked non‐specific protein binding, and then slices were incubated with primary antibodies against PKM2, LDHA, SOX2, c‐MYC, CD44 and CD133 at 4°C overnight. Finally, the images were observed using an optical microscope, collected, saved and analysed using CaseViewer and Aipathwell software.

### 2.3 Cell Culture

Human HCC cell lines, Huh7, HepG2 and PLC/PRF/5, were purchased from Wuhan Punosei Life Technology Co. Ltd. (Wuhan, China). Huh7 cells were cultured in Dulbecco's modified Eagle's culture medium supplemented with 10% foetal calf serum (FCS) (Gibco, Carlsbad, CA, USA), 100 U/mL penicillin, and 100 g/mL streptomycin. HepG2 and PLC/PRF/5 cells were cultured in Eagle's minimum essential medium (MEM) (Gibco, Carlsbad, CA, USA), adding 10% FCS, 100 U/mL penicillin, and 100 g/mL streptomycin; all cell lines were cultured in a humid environment at 37°C and 5% CO_2_.

### 2.4 Generation, Construction and Transfection of HBx‐Expressed or Interfered Vectors, as Well as Interfered PKM2 Vectors

The full‐length *HBx* gene with a Flag tag, short hairpin RNA (shRNA), and PKM2 shRNA were inserted into the lentivirus pLV[Exp]‐EGFP‐Puro vector, and the *HBx* gene was identified by PCR, restriction endonuclease and DNA sequencing. The HBx expression vector was named pLV‐HBx‐FLAG, the interfering HBx expression vector was named pLV‐shHBx, and the interfering PKM2 expression vector was named pLV‐shPKM2. Huh7 and HepG2 cells were infected with pLV‐HBx vectors, and pLV‐shHBx vectors were infected with PLC/PRF/5 cells, and stable cell clones were screened with puromycin. Cells stably expressing HBx were named Huh7‐HBx‐Flag and HepG2‐HBx‐Flag, and cells that stably interfered with HBx were named PLC/PRF/5‐shHBx. pLV‐shPKM2 vectors were used to infect Huh7‐HBx‐Flag and HepG2‐HBx‐Flag cell lines, respectively. The transfection rate and transfection effect of the expressing or interfering vectors were verified by fluorescence microscopy and Western blotting, respectively.

### 2.5 Simple Western Size Assay (Wes)

Simple Western Size Assays (Wes) are capillary‐based automated immunoassays. After the antibodies were diluted, the protein samples and reagents were configured according to the instructions, and all were added to the Wes board, which automatically completed the separation, fixation and detection of the protein samples with target‐specific antibodies and captured the data as a chemiluminescence image of the capillary electrophoresis. The Compass software then analyses the images, processes all the data, and provides the data. Wes was used to detect the expression of HK2, PKM2, LDHA, OCT4, c‐MYC, KLF4, CD44 and CD133 proteins in HCC tissues and cells. The assay antibodies of HK2 (1:200), PKM2 (1:100), LDHA (1:250), KLF4 (1:50), OCT4 (1:50), c‐MYC (1:50), CD133 (1:100), CD144 (1:100) and GAPDH (1:1000), all of which were purchased from Proteintech Group Inc. (Wuhan, China).

### 2.6 Western Blotting Analysis

After pLV‐HBx and pLV‐shHBx vectors were infected with Huh7, HepG2 and PLC/PRF/5, respectively, Western blotting was used to detect the expression of HBx, Flag, Akt, phosphorylated‐AKT (Ser473) [p‐Akt(Ser473)], PI3K and GAPDH proteins. The assay antibodies of HBx, Akt and PI3K (1:500) (Abcam, UK); p‐Akt (Ser473) (Cell Signalling Technology, US); GAPDH and FLAG tag (1:2000) (Proteintech Group Inc., Wuhan, China).

### 2.7 Real‐Time Quantitative PCR

Total RNA was extracted using an Eastep Super Total RNA Extraction Kit (Promega, Shanghai, China). cDNA synthesis was performed using the PrimeScript cDNA synthesis kit (Takara, Beijing, China). Quantitative reverse transcription PCR was performed using ChamQ Universal SYBR qPCR Master Mix (Vazyme, Nanjing, China). The reaction solution contained the cDNA template, 10 μM forward and reverse primers, and 2 × ChamQ Universal SYBR qPCR Master Mix. The primer sequences are described as follows: HBx, 5′‐CTCAGCAATGTCAACGACCG‐3′ (Forward primer) and 5′‐GCGCAGACCAATTTATGCCT‐3′ (Reverse primer); Flag, 5′‐AAAGACCATGACGGTGATTAT‐3′ (Forward primer) and 5′‐GagtCCGCGtaaagagagagg‐3′ (Reverse primer); HK2, 5′‐GCCAGAGCATCCTCCTCAAGTG‐3′ (Forward primer) and 5′‐TCACCACAGCAACCACATCCAG‐3′ (Reverse primer); PKM2, 5′‐TTGCCTGCTGTGTCGGAGAAG‐3′ (Forward primer) and 5′‐CagatGCCTTGCGGatGaatGA‐3′ (Reverse primer); OCT4, 5′‐GAGAACCGAGTGAGAGGCAACC‐3′ (Forward primer) and 5′‐CTGGGCGATGTGGCTGATCTG‐3′ (Reverse primer); KLF4, 5′‐AGAGACCGAGGAGTTCAACGATC‐3′ (Forward primer) and 5′‐GACGACGAAGAGGAGGCTGAC‐3′ (Reverse primer); c‐MYC, 5′‐GTCTGGATCACCTTCTGCTGGAG‐3′ (Forward primer) and 5′‐GCTGCGTAGTTGTGCTGATGTG‐3′ (Reverse primer). The amount of gene amplification product was normalised with respect to the amount of internal control agent (GAPDH).

### 2.8 Flow Cytometry Assay

Cells were maintained at 1 × 10^6^ cells/mL, and 5 μL Human TruStain FcX (Biolegend, USA) was added to 100 μL cell suspension and mixed for 10–15 min at room temperature. CD133 and CD44 fluorescent dye antibodies were added to 5 μL (Elabscience, Wuhan, China), gently mixed, and incubated at room temperature for 30 min. After incubation, 0.5–1 mL of Cell Staining Buffer (Biolegend, USA) was inserted into light‐repellent centrifuge tubes and centrifuged at 1500 rpm two times for 10 min. The data were collected by flow cytometry and analysed using FlowJo software.

### 2.9 Detection of Glucose Metabolism and the Product Concentration of Metabolism

Glucose consumption and pyruvate concentration were measured using a kit (Solarbio Science & Technology, Beijing, China); lactic acid concentration was detected using a kit (Nanjing Jianjieng Bioengineering Institute, Nanjing, China); and ATP levels were measured using a kit (Beyotime Biotechnology, Shanghai, China).

### 2.10 Protein Localization and Expression Were Observed by Laser Confocal Microscopy

After the cells were immobilised with 4% paraformaldehyde, 500 μL of QuickBlock immunostain blocking solution (Beyotime Biotechnology, Shanghai, China) was added to cell culture dishes specifically designed for laser confocal microscopes for 15 min. The blocking solution was permeable without additional permeability. Incubation was performed with PKM2 and OCT4 antibodies for 18 h, followed by the addition of donkey anti‐rabbit IgG H&L Alexa Fluor 647 (Abcam, UK) for 1 h, followed by the addition of an anti‐fluorescence quenching tablet containing DAPI (Beyotime Biotechnology, Shanghai, China) and a lucifuge seal for 20 min. Finally, the cells were observed under a ZEISS LMS800 laser confocal microscope (ZEISS, Germany).

### 2.11 Co‐Immunoprecipitation (Co‐IP) Assay

Cells were harvested using a lysis buffer (Sangon Biotech, Shanghai, China). Cell lysates (500 μg) were prepared and incubated overnight at 4°C with OCT4 (Abcam, UK) or rabbit IgG (Beyotime Biotechnology, Shanghai, China). The samples were then incubated with Protein A/G Magnetic Beads (Med Chem Express, USA) at 4°C for 2–4 h and buffered for Western blotting, and co‐immunoprecipitation (Co‐IP) cell lysate (Beyotime Biotechnology, Shanghai, China) was washed five to eight times and boiled to denature the protein. The protein samples were subjected to Western blotting analysis, incubated with OCT4 and PKM2 antibodies (Proteintech Group Inc., Wuhan, China), and secondary antibodies (Abcam, UK) with light and heavy chain reactions were used, and Co‐IP analysis was performed.

### 2.12 Stem Cell Pellet Experiment

The cells were digested with trypsin, washed, centrifuged with PBS, and suspended in serum‐free medium with the following components: DMEM/F‐12 (Gibco, USA) containing 20 ng/mL recombinant epidermal growth factor (EGF) and b‐fibre growth factor (b‐FGF) (Peprotech, USA) as well as 1× B27 and 1× N2 supplements (Gibco, USA). The cells were then inoculated at a controlled and appropriate density into a 6‐well plate of ultra‐low adhesion cells (Corning, USA). After 7–14 days of culture, the number of spheres per well was recorded and calculated.

### 2.13 Scratch Healing Experiment

Huh7 and HepG2 cells were transfected with HBx‐expressed vectors, and PLC/PRF/5 cells were transfected with shHBx vectors. Cells were seeded at a density of 5 × 10^4^ cells/well. Three parallel lines were drawn along the lower edge of the six‐well plate with a marker pen, separated by a distance of 1 cm. The scratch on the aperture of the cell was aligned perpendicular to the three horizontal lines located at the bottom. Subsequently, the medium was replaced every 24 h, and the cells that migrated to heal the scratch were observed at 0, 24 and 48 h, respectively, under an inverted microscope.

### 2.14 Soft Agar Colony Formation Test

The clonal potential of cells was evaluated using soft agar to form a semi‐solid culture medium. Briefly, a layer of 0.5% agar was first laid in a six‐well plate, and then the appropriate number of cells was mixed with 0.3% soft agar to form the upper gel. Finally, 200–500 μL of complete medium was added. The cells were supplemented with 200–500 μL complete medium every 3 days and incubated at 37°C and 5% CO_2_ for 14 days, and the number of clone spheroids formed was recorded and analysed.

### 2.15 Statistical Analysis

The experiment was repeated three times for each group. Aipathwell software was used to analyse the ratio of positive cells in the immunohistochemistry results, and automatic protein quantitative analysis was performed using the Wes Compass software to select the appropriate exposure time according to the difference in protein expression abundance and obtain Wes bands and corresponding grey values. Western blotting and other experiments were analysed using ImageJ image software, and GraphPad Prism Chinese software (version 9.0) was used to draw graphs and carry out statistical analysis. The measurement data of experimental data were represented as mean ± standard deviation ($\bar{x}\pm s$), and comparisons between two groups were analysed by *t* test or chi‐square test. Comparison between multiple groups was analysed by ANOVA, correlation analysis of two variables was analysed by correlation analysis, and *p* < 0.05 was considered statistically significant.

## 3 Results

### 3.1 The Warburg Effect Key Enzymes and CSC‐Related Proteins Were Highly Expressed in HCC Tissues

**FIGURE 1:**
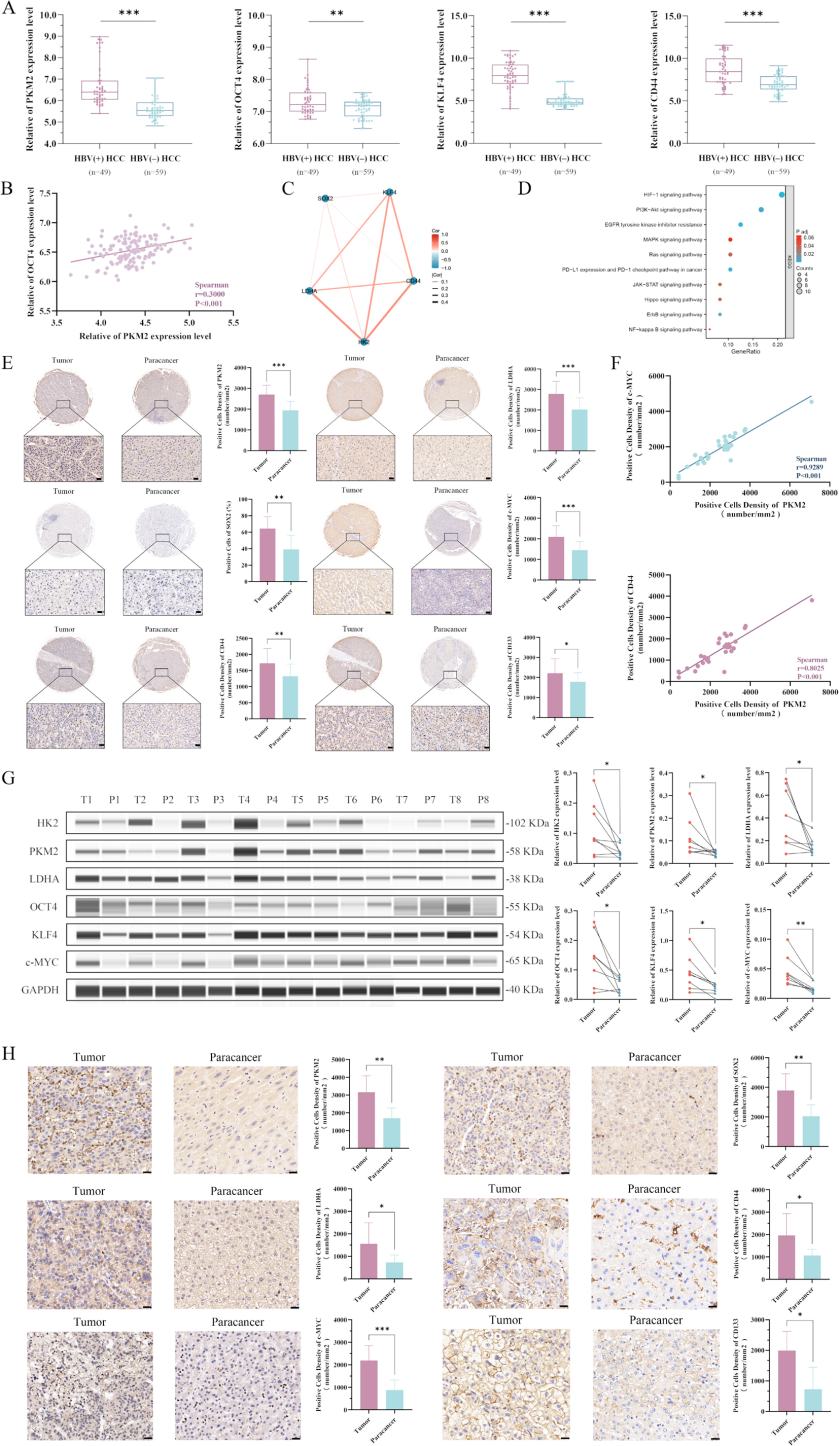
Correlation between HBV and the key enzymes of the Warburg effect and cancer stem cell reprogramming factors, as well as expression of PKM2 and cancer stem cell markers in cancer tissues and paracancerous tissues, was analysed by biological information. (A) 108 samples were collected from the GEO database, of which 49 were HBV infection‐causing HCC and 59 were other causes causing HCC. The expression of the key enzymes of the Warburg effect, cancer stem cell reprogramming factors and markers in HCC caused by HBV infection and HCC caused by other causes were analysed by protein expression differences. (B) The TCGA database collected 128 HCC tissue samples caused by HBV infection, and analysed the correlation between OCT4 and PKM2 in HCC caused by HBV infection. (C) Interaction between the key enzyme of the Warburg effect, cancer stem cell reprogramming factor and stemness marker. (D) Enrichment analysis of the key enzymes of the Warburg effect, cancer stem cell reprogramming factors and markers related to signalling pathways. (E) Expression of the key enzymes of the Warburg effect, cancer stem cell reprogramming factors and markers in the tissue chip of 30 HCC patients' cancer tissue samples and corresponding paracancerous tissues. (F) Correlation analysis of PKM2 and c‐MYC, PKM2 and CD44 in 30 HCC tissues. (G) Wes test results of cancer tissue specimens and corresponding paracancerous tissues of eight HCC patients. (H) Immunohistochemical results of cancer tissue samples and corresponding paracancerous tissues from eight HCC patients. *p < 0.05, **p < 0.01, ***p < 0.001.

The analysis displayed in the GEO database indicated that the expressions of the Warburg effect key enzyme PKM2, CSC reprogramming factors OCT4 and KLF4 and cancer stemness marker CD44 in HBV(+) HCC were higher than those of HBV(−) HCC, with statistical significance, *p* < 0.05 (Figure 1A). Subsequently, analysis of the TCGA database revealed that the expression level of OCT4 was positively correlated with PKM2 expression (*p* < 0.05) (Figure 1B). A protein interaction relationship was found between the key enzymes of the Warburg effect and the proteins associated with cancer stemness (Figure 1C). Moreover, the PI3K/Akt/mTOR signalling pathway, found in the rich concentration of related signalling pathways, suggested that this signalling pathway may play a role in the regulation of key enzymes of the Warburg effect, cancer stemness reprogramming factors and cancer stemness markers (Figure 1D).

Cancerous tissues and corresponding paracancerous tissues of 30 HCC patients were prepared into tissue chips, and it was found that the expressions of PKM2, LDHA, c‐MYC, SOX2, CD44 and CD133 in cancerous tissues were significantly higher than those in corresponding paracancerous tissues (*p* < 0.05), with statistical significance (Figure 1E). We further analysed the expression of PKM2, c‐MYC and CD44 in cancerous tissues, which were positively correlated, *p* < 0.01, with statistical significance (Figure 1F). The tissue samples of eight HCC patients were collected, and automated protein quantitative analysis (Wes) was used to detect that the expression of the Warburg effect key enzymes HK2, PKM2 and LDHA, as well as cancer cell stemness reprogramming factors OCT4, KLF4 and c‐MYC proteins in cancerous tissues, were significantly higher than those in corresponding paracancerous tissues, with the difference statistically significant (*p* < 0.05) (Figure 1G). Immunohistochemical experiments were performed on the eight HCC tissues, and it was found that the expression of PKM2, LDHA, c‐MYC, SOX2, CD44 and CD133 in cancerous tissues was significantly higher than that in paracancerous tissues (Figure 1H). These results for clinical HCC tissues were consistent with those of bioinformatics analysis.

### 3.2 HBx Induces the Expression of Cancer Cell Stemness Reprogramming Factors and Stem Cell Markers in HCC Cells

**FIGURE 2:**
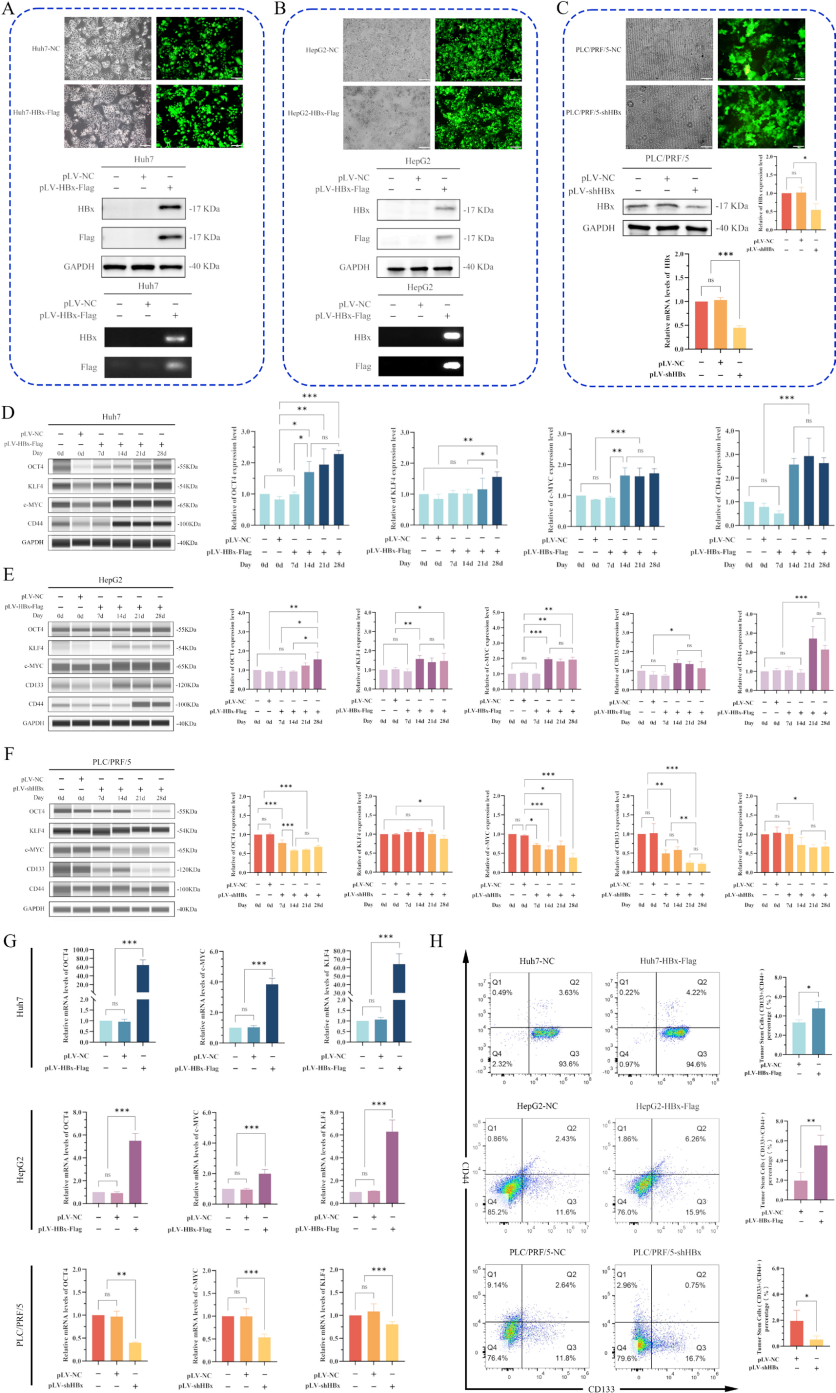
Construction of HBx‐expressed vectors and interfering HBx‐expressed vectors, and the effects of HBx on the expression of reprogramming proteins and markers of cancer stem cells. (A) After transfection of lentivirus pLV‐HBx‐Flag into the Huh7 cell line, the transfection efficiency was observed. Green fluorescence represented the cells successfully transfected. The fluorescence image was taken with a 10× objective microscope, and the scale was 100 μm. Western blotting and RT‐qPCR were used to detect the expression of HBx protein and Flag label protein of different groups in Huh7 cell lines, as well as mRNA transcription. (B) The green fluorescence expression of the HepG2 cell line after transfection with lentivirus pLV‐HBx‐Flag. The fluorescence images were taken with a 10× objective microscope and a 100 μm scale. Western blotting and RT‐qPCR were used to detect the expression of HBx protein and Flag label protein of different groups in HepG2 cell lines, as well as mRNA transcription. (C) Green fluorescence expression of lentivirus pLV‐shHBx transfected into the PLC/PRF/5 cell line. Fluorescence images were taken with a 10× objective microscope, and the scale was 100 μm. HBx expression in different groups of PLC/PRF/5 cell lines was detected by Western blotting, and the grey values of protein expression were statistically analysed after scanning. The mRNA of HBx in different groups of PLC/PRF/5 cell lines was detected by RT‐qPCR, and cycle threshold (Ct) value was used for statistical analysis. (D) Automated protein expression analysis (Wes) detected the expression of cancer stem cell reprogramming factors OCT4, KLF4, c‐MYC and cancer stem cell marker CD44 in Huh7 cell lines while transfected with HBx‐expressed vectors. The bar chart on the right showed statistical analysis of protein band grey values. (E) Wes was used to detect the expression of cancer stem cell reprogramming factors OCT4, KLF4 and c‐MYC, as well as cancer stem cell markers CD133 and CD44 in HepG2 cells while transfected with HBx‐expressed vectors. The bar chart on the right showed statistical analysis of protein band grey values. (F) Wes was used to detect the expression of cancer stem cell reprogramming factors OCT4, KLF4 and c‐MYC, as well as cancer stem cell markers CD133 and CD44 in PLC/PRF/5 cells while interfering with the expression of HBx. The bar chart on the right shows statistical analysis of protein band grey values. (G) RT‐qPCR was used to detect the transcriptional changes of OCT4, KLF4 and c‐MYC genes and the cycle threshold (Ct) values of mRNA were statistically analysed. (H) Flow cytometry was used to detect the proportion of cells with positive expressions of CD133 and CD44 on the cell surface. *p < 0.05, **p < 0.01, ***p < 0.001.

After infecting Huh7 and HepG2 cells with pLV‐HBx‐Flag lentivirus, Huh7 and HepG2 cell lines stably overexpressing HBx were established. At the same time, PLC/PRF/5 cells, which expressed HBx protein, were selected and transfected with pLV‐shHBx lentivirus (Figure 2A–C). Wes was used to analyse the change of cancer stemness cell reprogramming factors and stem cell markers in HCC cells at different time points. The cancer cell stemness reprogramming factors OCT4, KLF4 and c‐MYC and the stem cell marker CD44 were significantly increased in Huh7 cells on the 14th day after HBx overexpression (Figure 2D). In HepG2 cells, OCT4 and CD44 increased significantly on the 21st day after HBx overexpression, and OCT4 expression continued to be up‐regulated on the 28th day. KLF4, c‐MYC and CD133 increased significantly on the 14th day (*p* < 0.05) (Figure 2E). The expression of OCT4 and c‐MYC after PLC/PRF/5 cells were transfected with pLV‐shHBx began to decrease significantly on the seventh day and gradually decreased with the prolongation of time, which was statistically significant (*p* < 0.05). Similar changes were also observed in KLF4, CD133 and CD44 after interference with the expression of HBx, and their expression was significantly downregulated (Figure 2F). RT‐qPCR also showed that the transcription levels of the OCT4, KLF4 and c‐MYC in Huh7 and HepG2 cells were significantly enhanced following HBx overexpression. After interference with the expression of HBx, the transcription levels of the OCT4, KLF4 and c‐MYC genes were significantly decreased (*p* < 0.05) (Figure 2G). Flow cytometry was used to analyse the expression of stem cell markers CD133 and CD44 on the cell surface, and the percentage of CD133(+)/CD44(+) in Huh7 and HepG2 cells significantly increased after HBx overexpression. The number of CD133(+)/CD44(+) cells decreased significantly after interference with the expression of HBx (Figure 2H). The results indicated that overexpression of HBx could upregulate the expression of cancer cell stemness reprogramming factors and stem cell markers to varying degrees, and interfering with the expression of HBx could reduce the expression of cancer cell stemness reprogramming factors and stem cell markers.

### 3.3 HBx Promotes the Warburg Effect in HCC Cells

**FIGURE 3:**
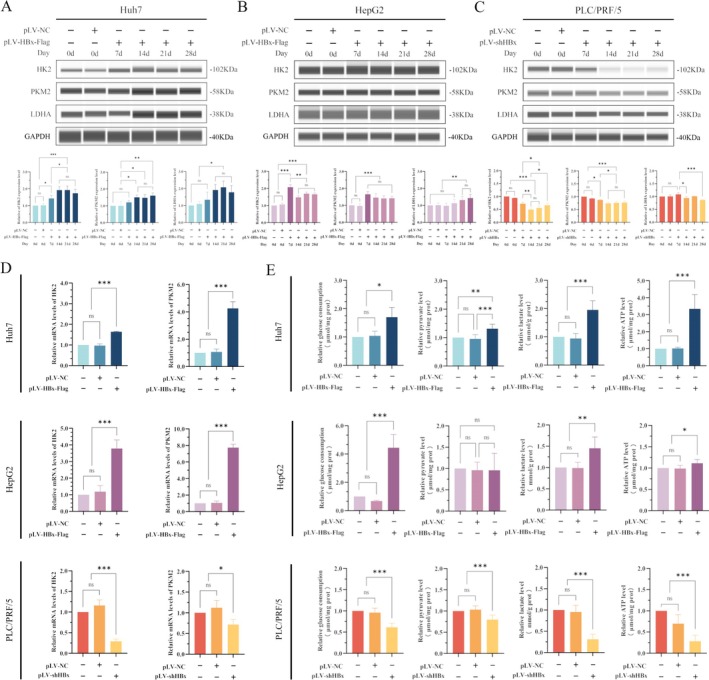
The effect of HBx on the expression and activity of key enzymes of the Warburg effect and the regulatory role in glucose metabolic reprogramming. (A, B) Wes was used to detect the expression of the Warburg effect key enzymes HK2, PKM2 and LDHA in Huh7 cells (A) and HepG2 cells (B) while transfected with HBx‐expressed vectors. The bar chart in below is a statistical analysis of protein band grey values. (C) Wes was used to detect the expression of the Warburg effect key enzymes HK2, PKM2 and LDHA in PLC/PRF/5 cells while interfering with the expression of HBx. The bar chart in below is a statistical analysis of protein band grey values. (D) RT‐qPCR was used to detect the effect of HBx on the transcription levels of HK2 and PKM2 genes in various HCC cells, and the Ct value of mRNA was statistically analysed. (E) The effect of HBx on glucose consumption, lactate, pyruvate and ATP production levels in various liver cancer cells was detected with a commercial kit. *p < 0.05, **p < 0.01, ***p < 0.001.

When Huh7 cells were transfected with HBx‐expressed vectors, the results indicated that the expression of HK2 began to up‐regulate on the 7th day after transfection, and the accompanying time increased gradually from the 14th day to the 28th day, which was statistically significant compared to the first day (*p* < 0.05). The expression of PKM2 and LDHA was slightly increased on 7th day, although not statistically significant, but began to be significantly up‐regulated on the 14th day(*p* < 0.05) (Figure 3A). The expression of HK2 and PKM2 in HepG2 cells was increased on the seventh day after transfection with HBx‐expressed vectors, compared to the first day, which was statistically significant (*p* < 0.05). The expression of LDHA also significantly increased at 21st day after transfection with HBx‐expressed vectors, compared to 1st day, with statistical significance (*p* < 0.05) (Figure 3B). The expression of HK2 in PLC/PRF/5 cells significantly decreased on the seventh day after transfection with pLV‐shHBx vectors, and the expression level continued to decrease with an increase in transfection time, which was statistically significant (*p* < 0.05). PKM2 protein expression began to be downregulated on the 7th day and gradually decreased after the 14th to the 28th day. LDHA was significantly downregulated on the 14th day, with statistical significance (*p* < 0.05) (Figure 3C). RT‐qPCR analysis also showed that the transcription levels of HK2 and PKM2 in Huh7 and HepG2 cells were significantly enhanced after transfection with HBx‐expressed vectors. After interference with the expression of HBx in PLC/PRF/5 cells, the transcription levels of the HK2 and PKM2 genes were significantly decreased (*p* < 0.05) (Figure 3D). With the up‐regulation of HBx protein, glucose consumption capacity, lactic acid and pyruvate production were increased in Huh7 cells, and ATP production was also up‐regulated compared with the normal Huh7 cells, which was statistically significant (*p* < 0.05). In HepG2 cells transfected with HBx‐expressed vectors, although pyruvate production did not change, glucose consumption, lactic acid, and ATP contents were significantly up‐regulated compared with the normal HepG2 cells, which had statistical significance (*p* < 0.05). After interference with the expression of HBx in PLC/PRF/5 cells, the results indicated that glucose consumption and pyruvate, lactic acid and ATP production levels in the PLC/PRF/5‐shHBx groups were significantly lower than those in the normal PLC/PRF/5 and negative control groups (PLC/PRF/5‐NC) (*p* < 0.001) (Figure 3E).

### 3.4 HBx Activates the PI3K/Akt/mTOR Signalling Pathway to Promote the Warburg Effect and Stimulates the Expression of CSC‐Related Proteins

**FIGURE 4:**
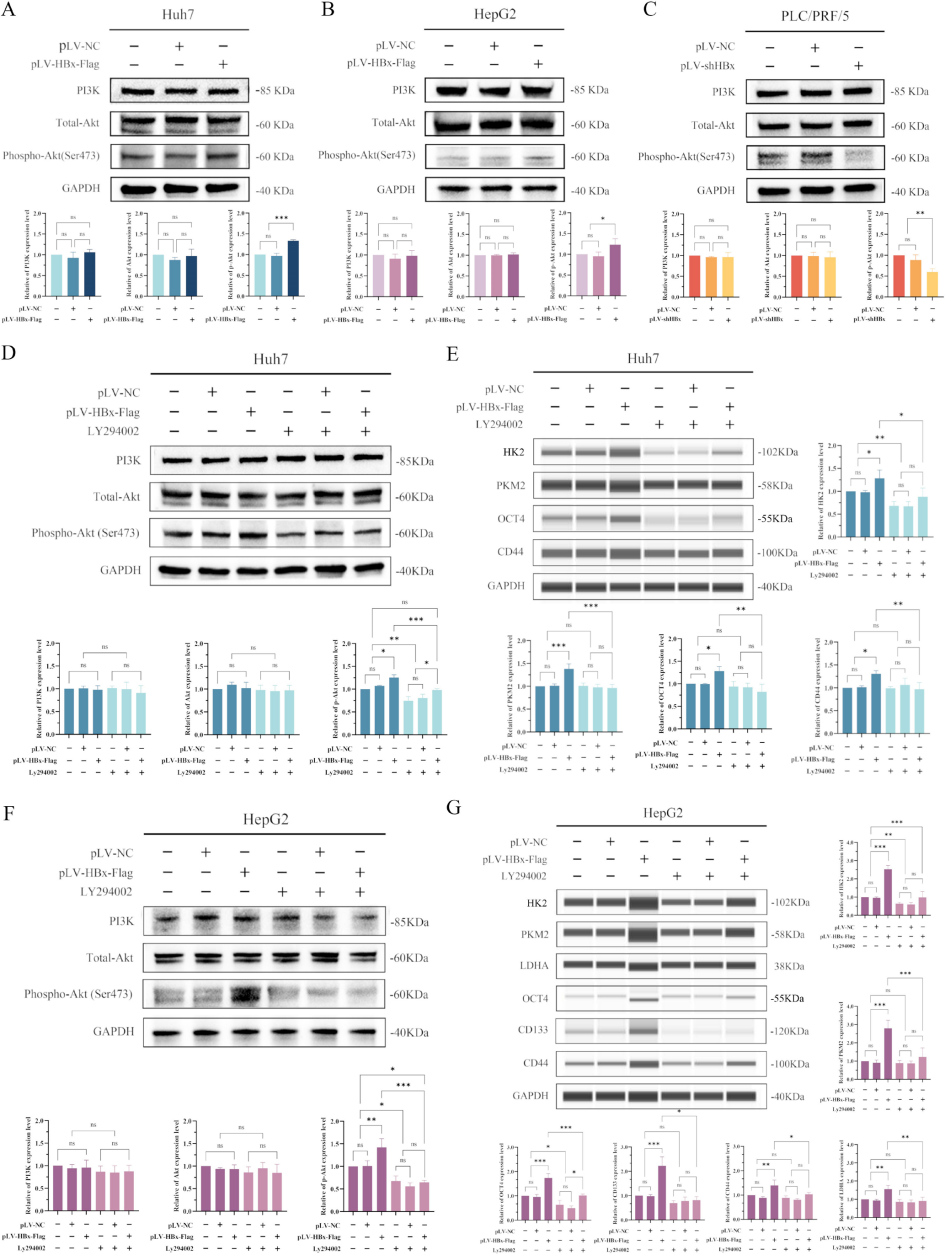
The role of HBx in the PI3K/AKT/mTOR signalling pathway and the expression of cancer stem cell reprogramming factors and markers in HCC cells. (A, B) Huh7 and HepG2 cells were transfected with HBx‐expressed vectors for 48 h, Western blotting was applied to measure the expression of PI3K/Akt/mTOR signalling pathway‐related proteins in Huh7 cells (A) and HepG2 cells (B). The bar chart below is a statistical analysis of the grey values of protein bands. (C) Western blotting was used to detect the expression of PI3K/Akt/mTOR signalling pathway related proteins in PLC/PRF/5 cell lines after interference with the expression of HBx. The sample size of proteins per lane was 80 ~ 100 μg. The bar chart below shows the statistical analysis of the grey values of protein bands. (D) Western blotting was applied to detect the expression of PI3K/Akt/mTOR signalling pathway‐related proteins in Huh7 cell lines after transfected with HBx‐expressed vectors, then Ly294002 was used to treat the cells for 24 h. The protein sample size per lane was 80 ~ 100 μg. The bar chart below is a statistical analysis of the grey values of protein bands. (E) Wes was applied to detect the expression of the Warburg effect key enzymes, cancer stem cell reprogramming factors and stemness markers in Huh7 cell lines after transfected with HBx‐expressed vectors then treatment with Ly294002 for 24 h. The bar chart in right and below is a statistical analysis of the grey values of protein bands. (F) Western blotting was used to detect the expression of PI3K/Akt/mTOR signalling pathway‐related proteins in HepG2 cells after transfected with HBx‐expressed vectors and then treated with Ly294002 for 48 h. The protein sample size per lane was 80 ~ 100 μg. The bar chart below is a statistical analysis of the grey values of protein bands. (G) Wes was applied to examine the expression of the Warburg effect key enzymes, cancer stem cell reprogramming factors and stemness markers in HepG2 cells after transfected with HBx‐expressed vectors then treatment with Ly294002 for 48 h. The bar chart on the right and below are statistical analyses of the grey values of protein bands. *p < 0.05, **p < 0.01, ***p < 0.001.

Western blotting experiments showed that while Huh7 and HepG2 cell lines were transfected with HBx‐expressing vectors, the results indicated that HBx could not change the expression of PI3K and total‐Akt, but the expression of phosphorylated‐Akt (Ser473) [p‐AKT(Ser473)] was significantly up‐regulated, the results being statistically significant (*p* < 0.05) (Figure 4A,B). After interference with the expression of HBx in the PLC/PRF/5 cells, the expression of PI3K and total‐Akt remained unchanged, but the expression of p‐AKT (Ser473) was significantly decreased (*p* < 0.01) (Figure 4C).

We selected Ly294002, an inhibitor of the PI3K/Akt/mTOR signalling pathway, to treat Huh7 and HepG2 cell lines which were transfected with HBx‐expressed vectors. The results indicated that Ly294002 could inhibit HBx‐stimulated expression of p‐AKT(Ser473), HK2, PKM2, LDHA, OCT4, CD133 and CD44. While the cells were treated with Ly294002, it was able to significantly downregulate the expression of these proteins in normal Huh7 and HepG2 cells (*p* < 0.05) (Figure 4D–G). These results indicated that HBx could up‐regulate the expression of key enzymes involved in the Warburg effect, stem cell reprogramming factors and cancer cell stemness markers by activating the PI3K/Akt/mTOR signalling pathway. Moreover, inhibition of the PI3K/Akt/mTOR signalling pathway by Ly294002 could reverse the expression of the key enzymes of the Warburg effect, stem cell reprogramming factors and cancer cell stemness markers which were promoted by the overexpression of HBx.

### 3.5 HBx Stimulates CSCs Reprogramming Through Activating PKM2

**FIGURE 5:**
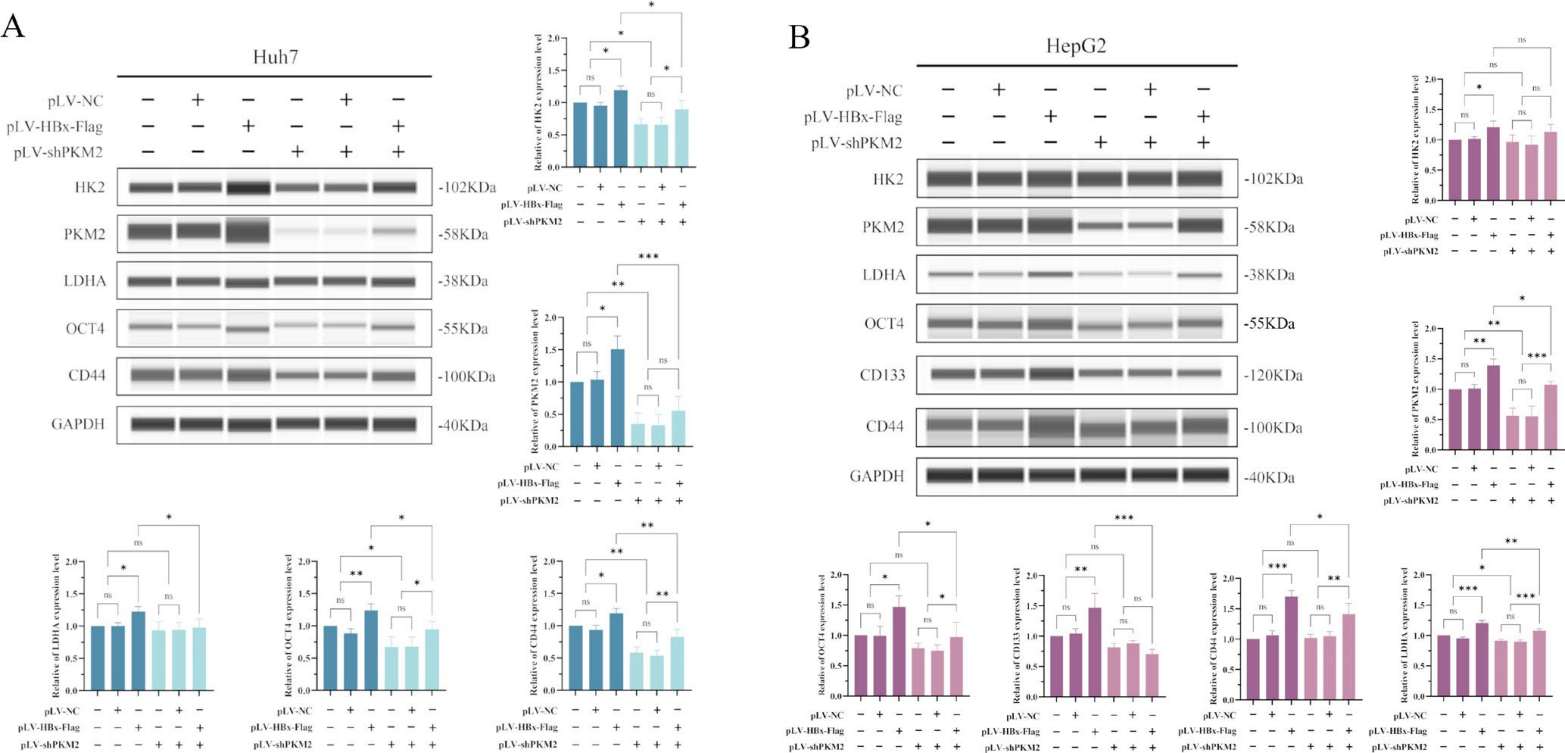
The effects of HBx and PKM2 on the expression of reprogramming factors and stemness markers in liver cancer cells. (A) Huh7 cells were transfected with HBx‐expressed vectors and constructed a stable HBx expression cell line, then the cells were transfected with shPKM2 vectors, Wes was used to detect the expression of HK2, PKM2, LDHA and OCT4 and CD44 in Huh7 cells. The bar chart on right and below is statistical analyses of protein band grey values. (B) HepG2 cells were transfected with HBx‐expressed vectors and constructed a stable HBx expression cell line, then the cells were transfected with shPKM2 vectors, Wes was used to detect the expression of HK2, PKM2, LDHA and OCT4, CD133 and CD44 in HepG2 cells. The bar chart below is a statistical analysis of the grey values of protein bands. *p < 0.05, **p < 0.01, ***p < 0.001.

We constructed a lentivirus that interferes with the expression of the PKM2 protein, and the normal group cells (Huh7 and HepG2), negative control vector group cells (Huh7‐NC and HepG2‐NC), and transfected with HBx group cells (Huh7‐HBx‐Flag and HepG2‐HBx‐Flag) were transfected with PKM2‐expressed vectors. After interfering with the expression of PKM2, the expression of key enzymes of the Warburg effect was significantly decreased. Although OCT4, CD133 and CD44 proteins in the normal groups (Huh7 and HepG2) and negative control groups (Huh7‐NC and HepG2‐NC) were not significantly changed; however, the expression of OCT4, CD133 and CD44 in Huh7‐HBx‐Flag and HepG2‐HBx‐Flag cells was significantly decreased with statistical significance (*p* < 0.05) (Figure 5A,B). These results indicated that interference with the expression of PKM2 in Huh7 and HepG2 cells, which overexpress HBx, could reverse the expression of CSC reprogramming factors and cancer cell stemness markers, which were up‐regulated by HBx. These results suggested that HBX promotes the reprogramming of CSCs by activating the activity of PKM2.

### 3.6 PKM2 Interacts With OCT4 Could Promote the Stem Cell Reprogramming of HCC Cells

**FIGURE 6:**
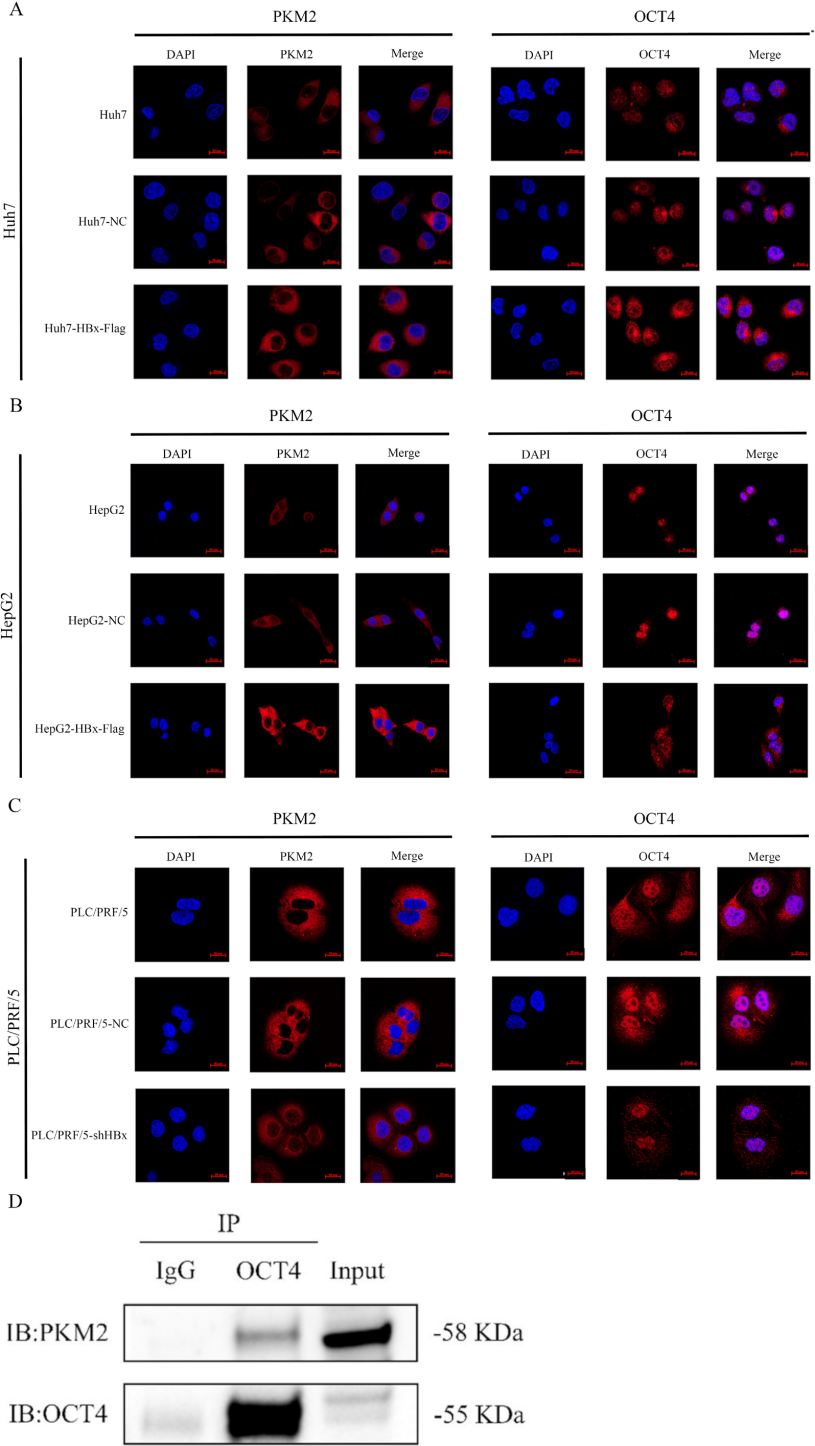
The influences of HBx on the expression of PKM2 and OCT4 in HCC cells, and the interaction between PKM2 and OCT4. (A, B) The localization and expression of PKM2 and OCT4 in Huh7 cells (A) and HepG2 cells (B) while transfected with HBx‐expressed vectors were observed by laser confocal microscopy, the objective microscope was 20×, the scale was 20 μm. (C) The localization and expression of PKM2 and OCT4 in the PLC/PRF/5 cells while interfered with the expression of HBx were observed by laser confocal microscopy with an objective microscope of 20× and a scale of 20 μm. (D) Co‐IP experiment was used to detect the interaction between PKM2 and OCT4 in HCC cells. The picture represents three repetitions of the experiment.

According to the results of the laser confocal microscopy, blue DAPI is the nuclear fluorescent dye, and red fluorescent dyes are PKM2 and OCT4, respectively. It could be observed from the merged images that PKM2 protein is mainly located in the cytoplasm, and a small amount is located in the nucleus. OCT4 is mainly located in the nucleus and cytoplasm. In Huh7‐HBx‐Flag and HepG2‐HBx‐Flag groups, the expression of PKM2 protein in the cytoplasm was significantly increased compared with that in the normal groups and pLV‐NC groups, and PKM2 protein formed a fuller shape around the nucleus. In the normal Huh7 and HepG2 groups and pLV‐NC groups, OCT4 protein was mainly expressed in the nucleus but was less expressed in the cytoplasm. The expression of OCT4 in the cytoplasm of Huh7‐HBx‐Flag and HepG2‐HBx‐Flag cells was significantly increased and mainly surrounded the nucleus (Figure 6A,B). Similar results were observed in PLC/PRF/5 cells, and the expression of PKM2 in the cytoplasm of PLC/PRF/5 cells with low HBx protein was significantly reduced. OCT4 protein was expressed in the cytoplasm of PLC/PRF/5 cells and around the nucleus; however, the expression of OCT4 in the cytoplasm was significantly reduced after interference with the expression of HBx (Figure 6C). The results of the laser confocal microscopy suggested that PKM2 and OCT4 were both expressed in the cytoplasm and nucleus, the locations of PKM2 and OCT4 may partially overlap around and within the nucleus, and the expression levels of both are positively correlated with the expression of HBx. We incubated the OCT4 protein antibody with the protein lysate of HepG2 cells which overexpressed HBx overnight, and carried out Western blotting experiments with PKM2 antibody and OCT4 antibody. Both OCT4 and PKM2 were expressed in the immunoprecipitation group of protein samples pulled down by the OCT4 antibody, indicating the interaction of OCT4 and PKM2 in the cells (Figure 6D). These results suggested that the Warburg effect stimulates CSC reprogramming through the interaction of PKM2 and OCT4 proteins.

### 3.7 HBx Promotes Malignant Behaviours of HCC

**FIGURE 7:**
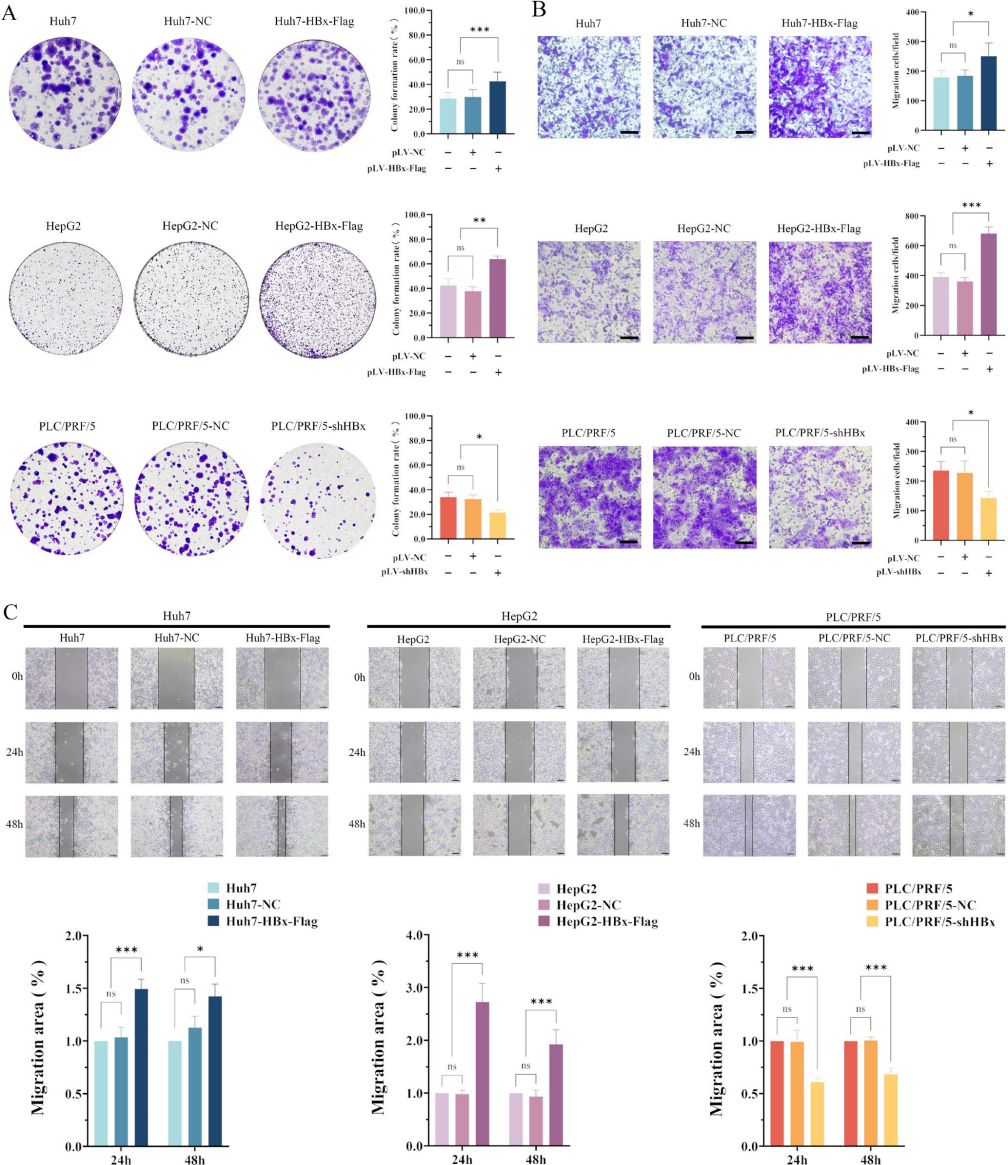
The role of HBx in clonal formation, invasion and migration of HCC cells. (A) Huh7 and HepG2 cells were transfected with HBx‐expressed vectors, and PLC/PRF/5 cells were interfering with the expression of HBx; the clonal formation ability of these cells was observed by plate cloning technique. After 14 days of continuous culture, the cellular colony was counted by crystal violet staining. The bar chart on right is a statistical analysis of the number of cloned cells. (B) Huh7 and HepG2 cells were transfected with HBx‐expressed vectors, and PLC/PRF/5 cells interfered with the expression of HBx. The invasive ability of these cells was detected by the Transwell test. The objective microscope was 10×, the scale was 100 μm and the bar chart on the right showed the statistical analysis of the number of invasive cells. (C) Huh7 and HepG2 cells transfected with HBx‐expressed vectors, and PLC/PRF/5 cells were interfered with the expression of HBx. These cells to repair the scratch were observed, and the repair ability was photographed under the microscope at 0, 24 and 48 h after the scratch, respectively. The objective microscope was 4× and the scale was 200 μm. The bar chart below is a statistical analysis of scratch repair. *p < 0.05, **p < 0.01, **p < 0.001.

The plate cloning ability test was used to detect cancer cell proliferation. The results showed that the number of colonies formed in the groups which transfected with HBx‐expressed vectors (Huh7‐HBx‐Flag and HepG2‐HBx‐Flag) was significantly higher than that in the normal HCC groups (Huh7 and HepG2) and the negative control vector groups (Huh7‐NC and HepG2‐NC) (*p* < 0.05). The number of colonies formed was significantly reduced in PLC/PRF/5 cells while interfered with the expression of HBx, indicating that the clonal formation ability of a single HCC cell was significantly decreased (*p* < 0.05) (Figure 7A). These results indicated that HBx was able to promote the clonal formation of a single HCC cell. The transwell assay revealed that the migratory ability was significantly increased in the Huh7‐HBx‐Flag and HepG2‐HBx‐Flag groups compared with the normal HCC groups (Huh7 and HepG2) and the negative control vector groups (Huh7‐NC and HepG2‐NC). However, the migratory ability was significantly reduced in the PLC/PRF/5‐shHBx groups compared to the normal PLC/PRF/5 groups and negative control vector groups (PLC/PRF/5‐NC). The numbers of Huh7‐HBx‐Flag and HepG2‐HBx‐Flag cells migrating through small pores increased significantly compared with normal Huh7 and HepG2 cells, with statistical significance (*p* < 0.05); after interfered with the expression of HBx, the number of PLC/PRF/5 (PLC/PRF/5‐shHBx) cells could be observed to be significantly reduced compared with normal groups (PLC/PRF/5) and negative control vector groups (PLC/PRF/5‐NC), with statistical significance (*p* < 0.05) (Figure 7B).

Scratch healing experiments were applied to observe the migration of HCC cells to repair the scratch distance. The results indicated that the migratory distance of the HBx‐expressed vector groups (Huh7‐HBx‐Flag and HepG2‐HBx‐Flag) was significantly increased compared with the normal HCC groups (Huh7 and HepG2) and the negative control vectors groups (Huh7‐NC and HepG2‐NC), with statistical significance (*p* < 0.05), as shown in Figure 7C. However, while interfered with the expression of HBx, it was observed that the migratory ability of PLC/PRF/5‐shHBx cell groups was significantly weakened compared with the normal PLC/PRF/5 cell groups and the negative control vectors groups (PLC/PRF/5‐NC), and the difference in motility was more significant after culturing for 48 h, with statistical significance (*p* < 0.05) (Figure 7C). These experimental results suggested that high expression of HBx could significantly promote the formation of cell clones and the migratory ability of liver cancer cells and also significantly enhance the scratch repair ability, thus increasing the metastatic ability of HCC. However, the malignant behaviours of HCC cells were significantly decreased after inhibiting the expression of HBx. These results showed that HBx played an important role in the malignant behaviours of HCC cells.

### 3.8 HBx Induces HCC Cells to Generate Cancer Stem/Progenitor Cells

**FIGURE 8:**
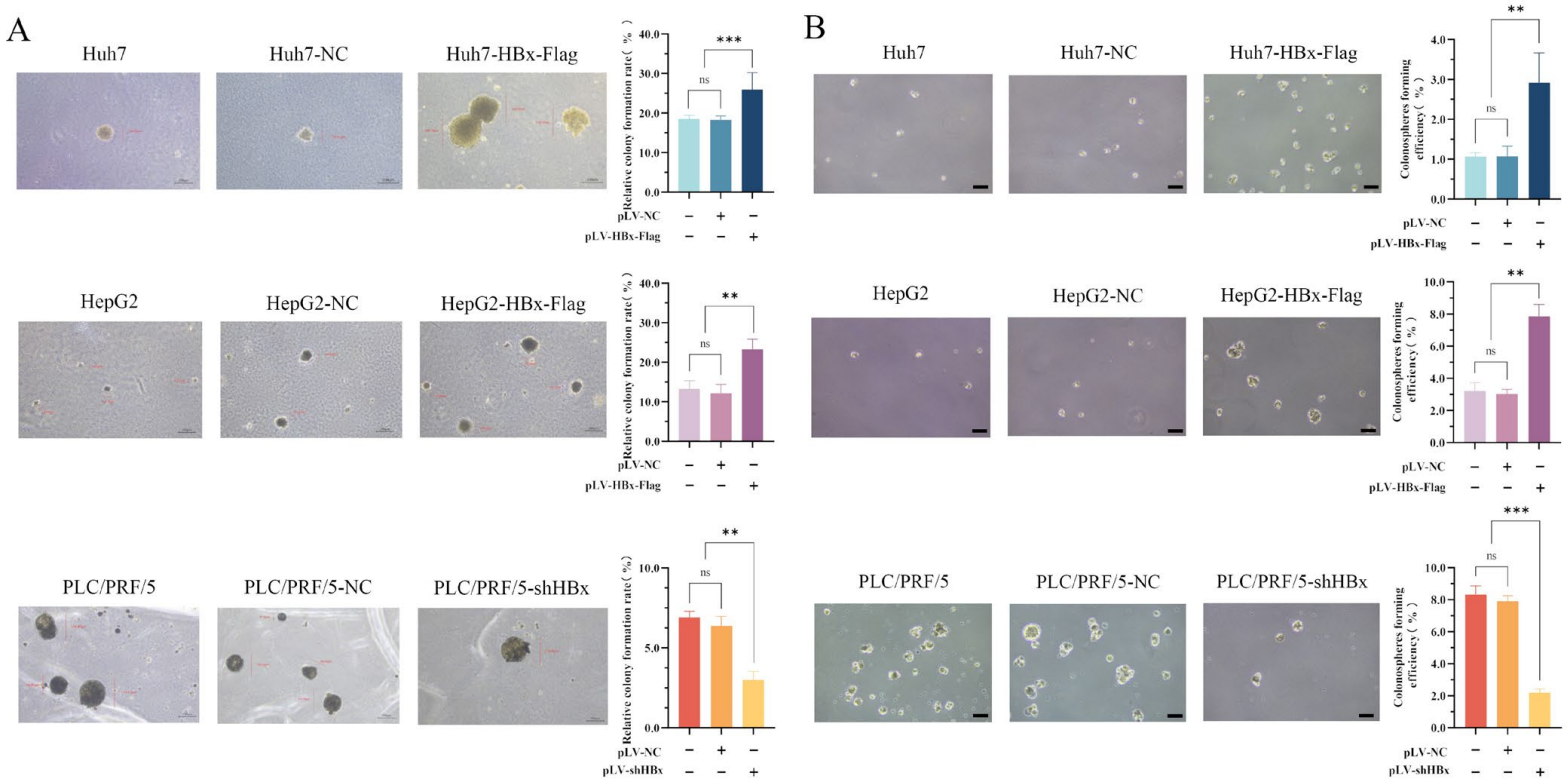
The influences of HBx on HCC stem cell generation. (A) Huh7 and HepG2 cells were transfected with HBx‐expressed vectors, and PLC/PRF/5 cells interfered with the expression of HBx. The growth of these cells clonal spheres was observed by the soft agar culture technique. After 14 days of culture, the clones were photographed with an objective microscope that was 4×, and the scale was 200 μm. The bar chart on the right is a statistical analysis of the number of cell clones formed. (B) Huh7 and HepG2 cells were transfected with HBx‐expressed vectors, and PLC/PRF/5 cells interfered with the expression of HBx. These cells were suspended and grown into stem cell clones in serum‐free medium. After 14 days of culture, the cell clones were photographed with a microscope. The objective microscope was 4×, and the scale was 200 μm. The bar chart on the right is a statistical analysis of the number of cell clones formed. *p < 0.05, **p < 0.01, ***p < 0.001.

The soft agar colony formation experiment was used to observe the generation of stem cells. In the present study, microscopy was used to observe colony formation by HCC cells. The results indicated that the diameter of the clone spheroids in the soft agar of normal HCC groups (Huh7 and HepG2) and the negative control vector groups (Huh7‐NC and HepG2‐NC) were approximately 100–200 μm, but in the transfected HBx‐expressed vector groups (Huh7‐HBx‐Flag and HepG2‐HBx‐Flag), the diameter of the clone spheroids could reach approximately 300 μm; the number of clones was also significantly increased, and the clone formation rate was also significantly increased compared to normal HCC groups and negative control vector groups (*p* < 0.01). These results indicated that overexpression of HBx in Huh7 and HepG2 cells significantly increased the cloning ability of single LCSCs compared with the normal groups (Huh7 and HepG2) and the negative control vector groups (Huh7‐NC and HepG2‐NC). However, while interfering with the expression of HBx, although the diameter of the clone of PLC/PRF/5 cells did not change significantly, the number of formed clones significantly decreased, indicating that the cloning ability of individual LCSCs was significantly reduced (*p* < 0.01) (Figure 8A).

We used serum‐free medium with appropriate cell growth factors to suspend LCSCs in a low‐adhesion culture plate and calculated the cell pellet formation rate of LCSCs. After 10 days of serum‐free suspension culture, the normal HCC groups (Huh7 and HepG2) and the negative control vector groups (Huh7‐NC and HepG2‐NC) were observed to form smaller stem cell clones under a microscope. In the cells transfected with HBx‐expressed vectors groups (Huh7‐HBx‐Flag and HepG2‐HBx‐Flag), the stem cell clones were larger in volume and better presented a complete and smooth spherical shape, and the number of stem cell clones was more significant compared to the normal HCC groups and the negative control vector groups, with statistical significance (*p* < 0.01). Compared with the interfered expression of HBx (PLC/PRF/5‐shHBx) groups, the normal PLC/PRF/5 groups and the negative control vectors groups (PLC/PRF/5‐NC) had a larger volume of stem cell clones; more abundant cells could be observed in the clones; and the number of stem cell clones was significantly higher. The results showed that the cell pellet formation rate of stem cells in the PLC/PRF/5‐shHBx groups was significantly decreased, which was statistically significant (*p* < 0.01) (Figure 8B). These results implied that HBx was able to induce partial transformation of the Huh7, HepG2 and PLC/PRF/5 cell lines into LCSCs and could promote the cell pellet‐forming ability of LCSCs after overexpression of HBx, while interfering with the expression of HBx was able to weaken the stem cell characteristics of LCSCs. These findings demonstrated that HBx has the biological function for promoting the generation of LCSCs.

## 4 Discussion

HCC is a common and frequent malignancy, and despite the emergence of various new therapies and significant clinical progress, the 5‐year overall survival rate remains disappointing [27]. The exact aetiology and molecular mechanism of HCC are still unclear, but HBV infection is the main cause of HCC [28]. Many studies have shown that HBx is a multifunctional regulator that plays a key role in the occurrence of HCC [29, 30]. Since drug resistance and relapse have been the main obstacles to the clinical treatment of HCC, many researchers have focused their attention on CSCs, which are a subgroup of poorly differentiated cancer cells with the ability to grow, regenerate and invade. Therefore, CSCs may be involved in cancer growth, invasion, metastasis, recurrence, chemotherapy resistance and other processes [31]. Therefore, it is important to explore the regulatory mechanism of HBx in CSC generation. OCT4, KLF4, SOX2, c‐MYC and other stem cell reprogramming factors play key roles in inducing stem cell formation, and many studies have shown that they can also promote the occurrence of CSCs in cancer and up‐regulate the expression of CSC markers [9, 26]. EpCAM, CD90, CD44 and CD133 are the most commonly reported CSC markers in HCC, and they are highly expressed in some tissues of patients with HCC. Moreover, high expression of CSC markers is significantly correlated with cancer grade, stage, AFP serum level, malignancy potential, high recurrence rate and short overall survival [32, 33], and HBx induces hepatocarcinogenesis closely related with reprogramming of glucose metabolism [34, 35]. Therefore, this study further investigated whether HBx is correlated with the reprogramming of glucose metabolism and the generation of LCSCs.

To clarify the association between HBx and LCSC reprogramming factors. In this study, we first conducted bioinformatics analysis using a biological information database to analyse the differences in cancer cell stemness reprogramming factors and CSC markers in HBV(+) or HBV(−) HCC. The results showed that the expression levels of CSC reprogramming factors and stem cell markers in HBV(+) HCC tissues were significantly higher than those in HBV(−) HCC tissues. Tissue samples from 38 cases of clinical HCC patients were collected and analysed using immunohistochemical tests and automated protein quantitative analysis (Wes). The results showed that the expression levels of CSC reprogramming factors and stemness markers in HCC patient tissues were significantly up‐regulated compared to those in paracancerous tissues. Based on the results of clinical tissue analysis and bioinformatics analysis, we speculated that HBx promotes the generation of CSCs by regulating the expression of CSC reprogramming factors and stemness markers.

Activation of the PI3K/Akt/mTOR signalling pathway has been shown to be an important risk factor for early recurrence and poor prognosis in HCC patients [36], as this signalling pathway can regulate cell cycle, survival, metabolism, invasion and angiogenesis. Therefore, the disturbance of the PI3K/Akt/mTOR signalling pathway leads to the boost of growth and promotes the migration and proliferation of HCC cells [37]. In this study, using bioinformatics analysis, we found that the PI3K/Akt/mTOR signalling pathway may be correlated with CSC reprogramming factors. Mangiapane et al. [38] found that targeting the PI3K/Akt/mTOR signalling pathway could inhibit the generation of colorectal CSCs and counteract anti‐EGFR treatment resistance. Recent studies in our laboratory have found that the PI3K/Akt/mTOR signalling pathway could also promote the occurrence of the Warburg effect in HCC, suggesting a significant correlation between the Warburg effect and LCSCs generation. Previous studies have reported that human embryonic stem cells (hESCs) exhibit typical metabolic changes, mainly in glycolytic mode, with lower levels of ATP and reactive oxygen species (ROS) than well‐differentiated cells because hESCs have fewer and more immature mitochondria, resulting in lower levels of oxidative phosphorylation [39]. However, research in the field of HCC is still unclear, so we conducted an in‐depth study on how HBx protein stimulates the occurrence of LCSCs and the relationship between HBx protein and the PI3K/Akt/mTOR signalling pathway and the Warburg effect.

In the present study, we selected the biological information database for analysis and found that the expression levels of key enzymes of the Warburg effect in HCC tissue samples displayed that the expression of these key enzymes in HBV(+) HCC tissues was significantly higher than in HBV(−) HCC tissues. The clinical HCC tissue samples were collected; immunohistochemical tests and Wes tests showed that the expression of key enzymes involved in the Warburg effect in HCC tissues was significantly higher than that in paracancerous tissues. Therefore, we speculated that HBx may regulate the expression of CSC reprogramming factors and stemness markers through activating the PI3K/Akt/mTOR signalling pathway and that the key enzymes of the Warburg effect are positively correlated with HBx protein. To further explore the regulatory mechanism of HBx on the generation of LCSCs, in vitro experiments with Huh7 and HepG2 cell lines, which do not express HBx, were selected and infected with HBx‐expressed vectors (pLV‐HBx‐Flag lentivirus). Huh7 and HepG2 cell lines stably overexpressing HBx protein were constructed. The pLV‐shHBx lentivirus was transfected into the PLC/PRF/5 HCC cell line, which expresses HBx, to construct a PLC/PRF/5 cell line that stably inhibited the expression of HBx. The results indicated that the expression of key enzymes of the Warburg effect, HK2, PKM2 and LDHA, began to increase significantly from the 7th day to the 14th day after being transfected with HBx‐expressed vectors in Huh7 and HepG2 cells. However, the expression of the CSC reprogramming factors OCT4, KLF4, c‐MYC and stemness markers CD44 and CD133 was significantly up‐regulated from the 7th day to the 21st day. After inhibiting the expression of HBx in PLC/PRF/5 cell lines, the expression of key enzymes of the Warburg effect, CSC reprogramming factors and stemness markers was found to be downregulated. RT‐qPCR results showed that the transcription levels of the Warburg effect key enzymes and CSC reprogramming factors were significantly increased after being transfected with HBx‐expressed vectors in HCC cells, while the transcription levels of key enzymes of the Warburg effect and CSC reprogramming factors were significantly decreased after interference with the expression of HBx. Flow cytometry results showed that the up‐regulation of HBx protein was able to increase the proportion of CD44(+)/CD133(+) cells, while interference with the expression of HBx could decrease the proportion of CD44(+)/CD133(+) cells.

Recently, evidence indicated that HBx was able to induce tumour stemness in HCC [40, 41]. HBx stimulates the expression of cellular OCT3/4 and MYC [42]; it is suggested that HBx has a function for promoting the generation of LCSCs. In the present study, the results showed that overexpression of HBx could promote the expression of key enzymes of the Warburg effect, CSC reprogramming factors and cell stemness markers, but this is only the regulation of key enzymes of the Warburg effect, and it cannot be proven that it has a promoting effect on the Warburg effect. Therefore, we examined the changes in glucose consumption and pyruvate, lactic acid and ATP production levels in HCC cells after overexpression or inhibition of HBx expression. The results showed that glucose consumption was significantly increased and the concentration of pyruvate, lactic acid and ATP was significantly enhanced in Huh7 cells while transfected with HBx‐expressed vectors. The results also showed that HepG2 cells, while transfected with HBx‐expressed vectors, were able to significantly increase glucose consumption and enhance the concentrations of lactic acid and ATP, but with no difference in pyruvate levels. This may be caused by differences between cells; HepG2 cells themselves have a high expression of LDHA; therefore, the expression level of LDHA significantly increases after HBx overexpression, which promotes the rapid conversion of pyruvate into lactic acid, resulting in a significant increase in the concentration of lactic acid and ATP. Glucose consumption was significantly reduced, and the concentrations of pyruvate, lactic acid and ATP were significantly decreased in PLC/PRF/5 cells while inhibiting the expression of HBx. Therefore, overexpression of HBx may promote the Warburg effect and increase the expression of CSC reprogramming factors and cell stemness markers. These results indicated that HBx could promote the reprogramming of glucose metabolism in hepatoma cells and stimulate the transformation of HCC cells into stem cells.

Although the research results have shown that HBx has the function of regulating the expression of reprogramming factors and stemness markers in CSCs, the influence of HBx on the malignant behaviours of HCC and the generation of LCSCs requires further exploration. Therefore, we used a cell scratch assay, Transwell assay, plate colony formation assay and soft agar colony formation assay to demonstrate the relationship between HBx and the malignant behaviours of HCC. The results indicated that when Huh7 and HepG2 cell lines were transfected with HBx‐expressed vectors, the scratch healing ability, cell migratory ability, and the clone formation rate in plate and soft agar were significantly increased. Based on the results of the non‐anchor‐dependent growth experiment, we speculated that LCSCs might exist in the Huh7 and HepG2 cell lines. Therefore, we used serum‐free cancer stem cell culture medium to culture cells that overexpressed HBx after 10 days to form larger and rounder tumour stem cell spheroids with more cells inside the cell clonal spheroids. In PLC/PRF/5 cells, while interfering with the expression of HBx, the healing ability of cells was decreased, the cell migratory ability was weakened, the clone formation rate of the plate and soft agar was significantly reduced, and the number and volume of cell clonal spheroids formed in serum‐free tumour stem cell culture medium were also significantly decreased. These results indicated that overexpression of HBx not only promoted the malignant behaviours of HCC but also increased the expression of CSC reprogramming factors and stemness markers and the generation of LCSCs.

Dysregulation of the PI3K/Akt/mTOR pathway is an important cause of tumourigenesis. Current studies have identified three types of PI3K. Many studies have shown that class I PI3K plays an important role in driving tumourigenesis and development. Class I PI3K generates phosphatidylinositol‐3,4,5‐triphosphate (PIP3) through activation of phosphorylation. Akt and 3‐phosphoinositide‐dependent protein kinase‐1 (PDK1) can be recruited to the plasma membrane, and PDK1 can activate phosphorylation to phosphorylate Akt protein. Thus, mTOR is activated to promote the transduction of signalling pathways [37, 43, 44, 45]. PI3K is highly expressed in HCC tissues, and PIK3CA, as one of the catalytic units of class I PI3K, upregulates the expression of HCC proliferation‐related proteins. In addition, high expression of PIK3CA is positively correlated with poor prognosis in patients with HCC [45]. According to other researchers, liver cancer is usually driven by the activation of PI3K signalling, and during the growth of HCC, PI3K signalling promotes a special metabolic mode, such as aerobic glycolysis, to obtain energy for cancer cells [46]. In the present study, bioinformatics analysis showed that the PI3K/Akt/mTOR signalling pathway is correlated with the Warburg effect and CSC reprogramming factors. Therefore, we explored the relationship between HBx and the PI3K/Akt/mTOR signalling pathway in vitro. Western blotting was used to detect the effect of HBx on the expression of PI3K/Akt/mTOR signalling pathway‐related proteins. The results indicated that after overexpression of HBx in HCC, the expression of PI3K/Akt/mTOR signalling pathway‐related proteins was significantly up‐regulated. However, the expression of PI3K/Akt/mTOR signalling pathway‐related proteins was significantly reduced in PLC/PRF/5 cells while inhibiting the expression of HBx. To explore whether the PI3K/Akt/mTOR signalling pathway affects the key enzymes that regulate the Warburg effect, the CSC reprogramming factor and stemness marker, Huh7 and HepG2 cells were transfected with HBx‐expressed vectors and treated with Ly294002. The results showed that after Ly294002 treatment, the expression of the PI3K/Akt/mTOR signalling pathway‐related proteins was significantly decreased, indicating that the PI3K/Akt/mTOR signalling pathway was successfully inhibited. The results also showed that the expression of key enzymes of the Warburg effect, CSC reprogramming factors, and cell stemness markers was significantly reduced, and the up‐regulation effects of overexpression of HBx on key enzymes of the Warburg effect, CSC reprogramming factors and stemness markers could be reversed. These results demonstrated that HBx could promote the Warburg effect and generation of LCSCs in HCC by activating the PI3K/Akt/mTOR signalling pathway.

The Warburg effect is a strange phenomenon in which cancer cells prefer glycolytic energy production even when the oxygen supply is sufficient. The activities of key rate‐limiting enzymes, including PKM2, LDHA and HK2, regulate glucose glycolysis. These factors influence the Warburg effect, which is significantly increased during HCC progression. Therefore, cancer cells can adapt to hypoxic environments for growth and invasion. Our study has found that changing the expression of HBx leads to the expression of key enzymes of the Warburg effect changing earlier than the changes in CSC reprogramming factor and cell stemness markers; therefore, we further explored the relationship between the Warburg effect and CSC‐related proteins. We transfected the lentivirus pLV‐shPKM2 into Huh7 and HepG2 cells which overexpress HBx. Wes results showed that the expression of OCT4, CD44 and CD133 was significantly decreased after interfering with the expression of PKM2. Moreover, overexpression of HBx could up‐regulate the expression of OCT4, CD44 and CD133, which could be reversed by inhibiting the expression of PKM2 in HCC cells. These results suggested that the key enzymes of the Warburg effect were positively correlated with cell stemness reprogramming factors and markers.

Pyruvate kinase isoenzyme type M2 is a subtype of the pyruvate kinase family, and PKM is a key glycolytic enzyme that was significantly up‐regulated during tumourigenesis [46]. PKM1 and PKM2 are splicing products of the same mRNA transcription product. The expression of pyruvate kinase isoenzymes depends on the metabolism of the different cells and tissues. PKM2 is expressed in early foetal tissues and is present in most adult tissues. The expression of PKM2 in skeletal muscle, heart, and brain was gradually replaced by PKM1. Cancer cells are characterised by abnormal proliferation; therefore, in the process of cancer initiation, the expression of pyruvate kinase isoenzymes can forebode the occurrence of cancer. In the brain, which originally expressed PKM1, and the liver, which originally expressed PKL, the pyruvate kinase isoenzyme decreased; instead, PKM2 was up‐regulated. In addition, PKM2 is positively correlated with malignancy in many tumours; therefore, the high expression of PKM2 protein has strong specificity in malignant tumours [46, 47, 48]. Previous studies have found that the cell stemness reprogramming factor OCT4 plays a crucial role in promoting stem cell generation because it acts as a master switch during differentiation and can regulate cells with pluripotent potential or is capable of generating pluripotent potential [49]. It has been reported that the absence of liver tissue‐specific PKL and the subsequent expression of PKM2 are the first steps in multi‐step carcinogenesis, and relevant experiments have been conducted in other cancers, suggesting that PKM2 may interact with OCT4 to a certain extent [50, 51]. However, it remains unclear whether PKM2 can synergistically regulate the occurrence of LCSCs by interacting with OCT4 in HCC. In the present study, the results indicated that the key enzymes of the Warburg effect were positively correlated with cancer stemness reprogramming factors and markers and further investigated whether PKM2 interacts with OCT4 in HCC cells. We used immunofluorescence to observe the location and expression of PKM2 and OCT4 in Huh7 and HepG2 cells after overexpression of HBx and PLC/PRF/5 cells after interfering with the expression of HBx; the results showed that PKM2 was mainly located in the cytoplasm, a small amount was located in the nucleus, OCT4 was mainly located in the nucleus, and a small amount was located in the cytoplasm. The expression of OCT4 and PKM2 in the cytoplasm was significantly up‐regulated after overexpression of HBx in HCC cells. However, while interfering with the expression of HBx, the high expression of OCT4 in the cytoplasm changed to a state mainly located in the nucleus, and the expression of PKM2 was significantly reduced, suggesting that PKM2 and OCT4 overlapped in the peri‐nucleus and in the cytoplasm. Co‐IP experiments showed that PKM2 could interact with OCT4, implying that PKM2 was able to regulate the role of OCT4 and induce reprogramming of liver cancer cells, thus promoting the generation of LCSCs.

**FIGURE 9:**
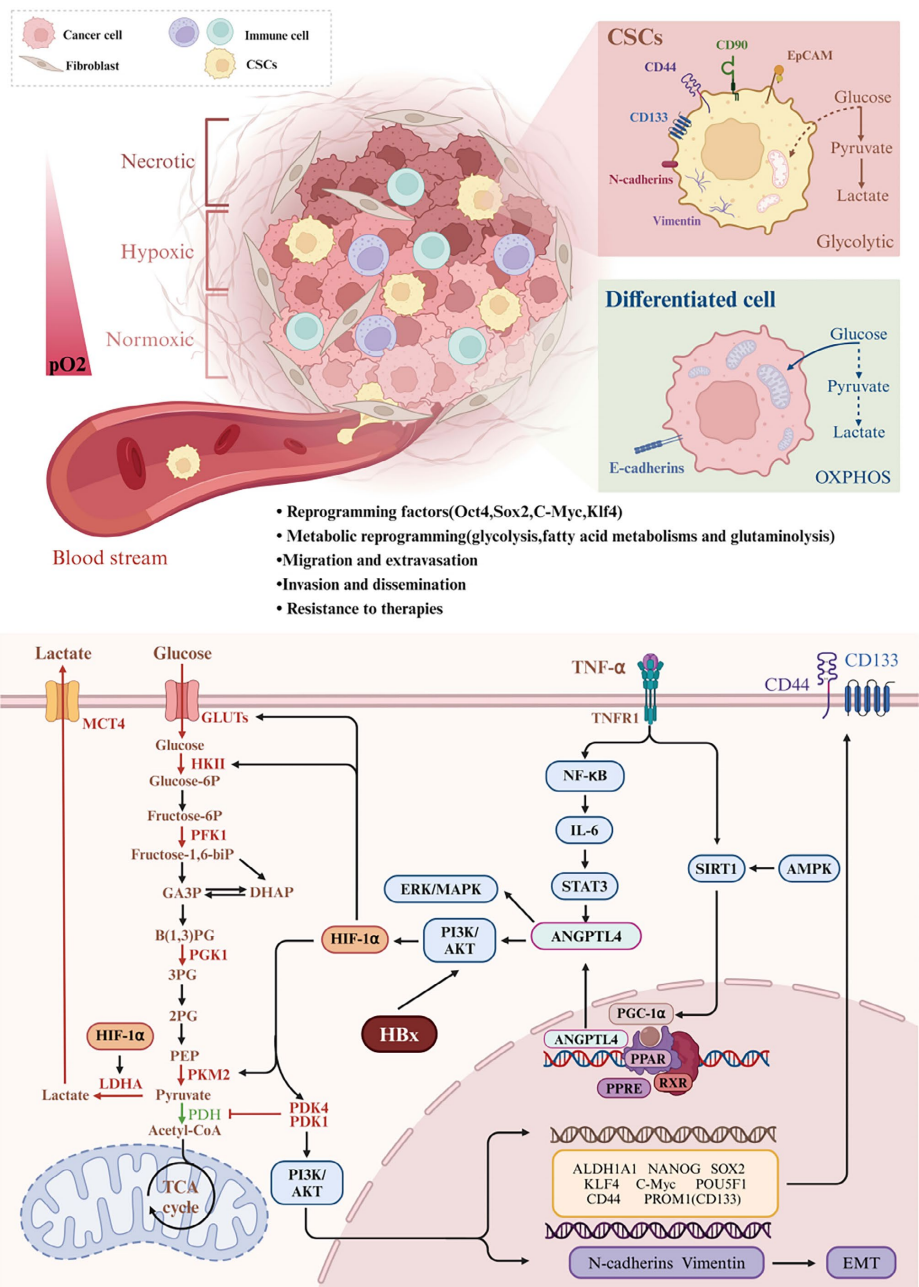
The heterogeneity of glucose metabolism in HCC tissue and the mechanism of HBx‐induced glucose metabolic reprogramming to promote the generation of HCC stem cells. The metabolic heterogeneity of HCC tissue and cancer stem cells was shown. Because there are many types of cancer cells and they have different metabolic modes, they are affected by the tumour microenvironment under different conditions, which affects the metabolic modes of different cells in cancer tissues. First of all, tumour cells near blood vessels exhibit oxidative phosphorylation metabolism due to sufficient nutrients such as oxygen and glucose. However, when a tumour extends deeper and grows farther away from blood vessels, resulting in insufficient nutrients, tumour cells may observe a shift to glycolytic metabolism. Aerobic glycolysis remains even when exposed to oxygen. In addition, the rapid proliferation of cancer cells close to blood vessels will cause oxidative stress, resulting in increased mitochondrial autophagy and the production of a large amount of lactic acid can also provide metabolic raw materials for cancer cells in distant blood vessels, so metabolic reprogramming will occur in cancer cells. Second, the presence of a small number of malignant liver cells in tumour tissue also favours aerobic glycolysis because of their naive mitochondria, which also helps CSCs to rapidly proliferate, metastasise and invade (shown in the above image). In the development process of HCC, HBx expressed after HBV infection in hepatocytes acts on the PI3K/Akt signalling pathway, which not only promotes the malignant transformation of hepatocytes but also continuously activates the PI3K/Akt signalling pathway and promotes the expression of key enzymes (PKM2, LDHA, HK2, etc.) which regulate aerobic glycolysis after the occurrence of HCC. This induces a reprogramming of glucose metabolism (Warburg effect). This study found that the Warburg effect can stimulate the generation of LCSCs, which leads to drug resistance and recurrence of liver cancer (shown in the below image). 3PG, 3‐phosphoglycerate; Akt, protein kinase B; ALDH1A1, acetaldehyde dehydrogenase 1A1; AMPK, AMP‐activated protein kinase; ANGPTL4, angiopoietin‐like protein 4; B(1,3)PG, 1,3‐bisphosphoglycerate; c‐MYC, cellular‐myelocytomatosis viral oncogene; CSCs, cancer stem cells; DHAP, dihydroxyacetone phosphate; EMT, epithelial‐mesenchymal transition; GA3P, glyceraldehyde3‐phosphate; GLUTs, glucose transporter; HIF‐1α, hypoxia‐inducible factor‐1α; HK‐II, hexokinase II; IL‐6, interleukin‐6; KLF4, Kruppel‐like factor4; LDHA, lactate dehydrogenase; MCT4, monocarboxylic acid transporter 4; NF‐κB. nuclear factor kappa‐B; PDH, pyruvate dehydrogenase complex; PDK1, pyruvate dehydrogenase kinase 1; PDK4, pyruvate dehydrogenase kinase 4; PEP, phosphoenolpyruvate; PFK1, 6‐phosphofructokinase1; PGC‐1α, peroxisome proliferator‐activated receptor gamma coactivator 1‐alpha; PGK1, phosphoglycerate kinase 1; PI3K, phosphatidylinositol 3‐kinase; PKM2, pyruvate kinase 2; POU5F1(OCT3/4), POU class 5 homeobox 1(Octamer‐binding transcription factor 3/4); PPAR, peroxisome proliferators‐activated receptors; PPRE, peroxisome proliferator response element; PROM1, CD133; RXR, retinoid X receptor; SIRT1, sirtuin 1; Sox2, sex determining region Y‐box 2; STAT3, signal transducer and activator of transcription 3; TCA cycle, tricarboxylic acid cycle; TNFR1, tumour necrosis factor receptor 1; TNF‐α, tumour necrosis factor‐α.

This study found that the HBx could regulate the Warburg effect and the generation of LCSCs, and the expression of HBx was positively correlated with the expression of key enzymes of the Warburg effect, cancer cell stemness reprogramming factors, and markers. HBx promotes the Warburg effect and generation of LCSCs by activating the PI3K/Akt/mTOR signalling pathway. This study revealed the regulatory role of HBx in the development and progression of HCC and found that HBx up‐regulates the Warburg effect by activating the PI3K/Akt/mTOR signalling pathway. The product of the Warburg effect is lactic acid; under the regulation of lactylase, lactic acid writes in the specific lysine of histone or non‐histone, and these proteins are lactated, affecting gene transcription and intracellular signal transduction. Many studies have found that lactylation is closely related to the generation of LCSCs [52, 53, 54]. The study found that HBx promotes the Warburg effect and stimulates the production of lactic acid, implying that the HBx‐lactylation axis can stimulate the generation of LCSCs. This study displayed the key regulatory molecules of drug resistance and relapse in the clinical treatment of liver cancer and found new evidence that PKM2 interacts with OCT4 and stimulates the initiation of LCSCs. We summarised the spatial heterogeneity of liver cancer tissue and the molecular mechanism of HBx inducing glucose metabolic reprogramming to promote the generation of LCSCs, as shown in Figure 9.

## 5 Conclusions

The expression of key enzymes of the Warburg effect, cancer cell stemness reprogramming factors and markers was significantly higher in HCC patients than in corresponding paracancerous tissues. The key enzyme of the Warburg effect is positively correlated with the expression of cancer cell stemness reprogramming factors and markers. HBx stimulates the generation of LCSCs and promotes HCC malignant behaviours. HBx enhances the Warburg effect by activating the PI3K/Akt/mTOR signalling pathway, thereby up‐regulating the expression of cancer cell stemness‐related proteins. HBx could promote the reprogramming of cancer cells by inducing glucose metabolic reprogramming of liver cancer cells. Lactic acid, the main product of the Warburg effect, stimulates the generation of LCSCs, which leads to liver cancer recurrence, drug resistance and metastasis, suggesting that targeting HBx and PKM2 is a new direction for solving the problems of HCC drug resistance and recurrence.

## Author Contributions

**Jinchen Liu:** investigation (equal), writing – original draft (lead). **Xueqin Wu:** investigation (equal), resources (equal), writing – original draft (equal). **Qiushi Yin:** formal analysis (equal), investigation (equal), resources (equal). **Luying Zhang:** formal analysis (equal), investigation (equal). **Kun Liu:** conceptualization (equal), formal analysis (equal), methodology (equal). **Kailin Huang:** formal analysis (equal), investigation (equal). **Junnv Xu:** conceptualization (equal), resources (equal). **Xiaowei Li:** formal analysis (equal), investigation (equal). **Bo Lin:** conceptualization (equal), project administration (equal). **Mingyue Zhu:** conceptualization (equal), project administration (equal), supervision (equal), writing – original draft (equal). **Mengsen Li:** conceptualization (lead), funding acquisition (lead), supervision (lead), writing – review and editing (lead).

## Ethics Statement

This study's subjects provided informed consent, and the collection and use of tissue specimens were approved by the Medical Ethics Committee of the First Affiliated Hospital of Hainan Medical University and the Second Affiliated Hospital of Hainan Medical University. The Institutional Ethics Committee of the Second Affiliated Hospital of Hainan Medical University and the institution review board number was No. LW2019312, and the protocol number was No. 2019‐043.

## Consent

All authors have read and agreed to publish this manuscript.

## Conflicts of Interest

The authors declare no conflicts of interest.

## Data Availability

All data generated or analyzed during this study are included in this manuscript.
